# Microbial community variations in adult *Hyalomma dromedarii* ticks from single locations in Saudi Arabia and Tunisia

**DOI:** 10.3389/fmicb.2025.1543560

**Published:** 2025-02-11

**Authors:** Myriam Kratou, Apolline Maitre, Lianet Abuin-Denis, Rachid Selmi, Hanène Belkahia, Abdullah D. Alanazi, Hattan Gattan, Bassam M. Al-Ahmadi, Abdullah F. Shater, Lourdes Mateos-Hernández, Dasiel Obregón, Lilia Messadi, Alejandro Cabezas-Cruz, Mourad Ben Said

**Affiliations:** ^1^Laboratory of Microbiology, National School of Veterinary Medicine of Sidi Thabet, University of Manouba, Manouba, Tunisia; ^2^ANSES, INRAE, Ecole Nationale Vétérinaire d’Alfort, UMR BIPAR, Laboratoire de Santé Animale, Maisons-Alfort, France; ^3^INRAE, UR 0045 Laboratoire de Recherches Sur Le Développement de L’Elevage (SELMET LRDE), Corte, France; ^4^EA 7310, Laboratoire de Virologie, Université de Corse, Corte, France; ^5^Animal Biotechnology Department, Center for Genetic Engineering and Biotechnology, Havana, Cuba; ^6^Department of Biological Sciences, Faculty of Science and Humanities, Shaqra University, Ad-Dawadimi, Saudi Arabia; ^7^Department of Medical Laboratory Sciences, Faculty of Applied Medical Sciences, King Abdulaziz University, Jeddah, Saudi Arabia; ^8^Special Infectious Agents Unit, King Fahad Medical Research Center, Jeddah, Saudi Arabia; ^9^Department of Biology, Faculty of Science, Taibah University, Madinah, Saudi Arabia; ^10^Department of Medical Laboratory Technology, Faculty of Applied Medical Sciences, University of Tabuk, Tabuk, Saudi Arabia; ^11^School of Environmental Sciences, University of Guelph, Guelph, ON, Canada; ^12^Department of Basic Sciences, Higher Institute of Biotechnology of Sidi Thabet, University of Manouba, Manouba, Tunisia

**Keywords:** *Hyalomma dromedarii*, microbiota, geographic variation, sex-specific differentiation, co-occurrence network analysis, microbial resilience, tick-borne pathogens

## Abstract

**Introduction:**

The camel-infesting tick, *Hyalomma dromedarii*, is a prominent ectoparasite in the Middle East and North Africa (MENA) region, critically impacting camel health and acting as a vector for tick-borne pathogens. Despite prior studies on its microbiota, the effects of geographic origin and sex on microbial community structure and functional stability remain poorly understood.

**Methods:**

To address this, we characterized the bacterial microbiota of *H. dromedarii* ticks collected from camels in Tunisia (TUN) and Saudi Arabia (SA) using 16S rRNA gene sequencing, microbial network analysis, and metabolic pathway prediction.

**Results:**

Our findings indicate a dominant presence of *Francisella* endosymbionts in Tunisian ticks, suggesting adaptive roles of *H. dromedarii* ticks in arid ecosystems. Keystone taxa, particularly *Staphylococcus* and *Corynebacterium*, were identified as central to microbial network structure and resilience. Moreover, network robustness analyses demonstrated enhanced ecological stability in the Tunisian tick microbiota under perturbation, indicative of higher resilience to environmental fluctuations compared to Saudi Arabian ticks. Additionally, functional pathway predictions further revealed geographically distinct metabolic profiles between both groups (Tunisia vs. Saudi Arabia and males vs. females), underscoring environmental and biological influences on H. dromedarii microbiota assembly.

**Discussion:**

These results highlight region-specific and sex-specific microbial adaptations in *H. dromedarii*, with potential implications for pathogen transmission dynamics and vector resilience. Understanding these microbial interactions may contribute to improved strategies for tick control and tick-borne disease prevention.

## 1 Introduction

Camel farming has long been a cornerstone of the Arabian Peninsula’s economy, a role further enhanced by recent development initiatives (Al-Deeb and Muzaffar, 2020). Today, the region supports a population of over 15 million camels, underscoring the livestock sector’s continued reliance on camel production (Al-Deeb and Muzaffar, 2020). In the United Arab Emirates (UAE), a vast desert area extending from Oman to Saudi Arabia, camels make up a significant portion of the fauna, with an estimated population of 459,000 (Perveen et al., 2020a). Furthermore, Douz, located in Tunisia and known as the “gateway to the Sahara,” is a crucial center for camel trade and commerce, highlighted by its prominent role as a major marketplace and weekly trading hub for camel and donkey owners and breeders (Demoncheaux et al., 2012). Despite its importance in Saudi Arabia and Tunisia, camel production faces challenges from pathogens including viruses, bacteria, parasitic protozoans, helminths and ticks, often exacerbated by transnational animal movements (Khalafalla, 2017).

The camel tick, *Hyalomma dromedarii* (Acari: Ixodidae), represents a significant threat to the health of camels due to its role as a vector for tick-borne diseases in both camels and humans (Walker et al., 2003; Perveen et al., 2022a). *Hyalomma dromedarii* exhibits a wide geographical distribution, spanning from North and Northwest Africa to Central and East Africa, as well as extending into the Middle East and Central and South Asia (Apanaskevich et al., 2008). Notably, it stands out as the most frequently encountered tick species infesting camels in Saudi Arabia (El-Azazy and Scrimgeour, 1997; Alanazi et al., 2019), asserting its dominance as the primary tick infesting camels across the MENA region (Alanazi et al., 2020; Perveen et al., 2021a). Furthermore, while predominantly inhabiting desert areas within Tunisia, *H. dromedarii* has also been observed on camels in the semi-arid regions of northern Tunisia, indicating its adaptability to varying ecological conditions (Elati et al., 2021). Apart from pathogenic microbes, *H. dromedarii* also hosts non-pathogenic microbes, including endosymbionts such as *Francisella*-like endosymbionts (FLEs) (Ghoneim et al., 2017; Azagi et al., 2017).

Tick species play a key role, with each species exhibiting unique adaptations that influence gut microbial communities (Alice et al., 2023; Tonk-Rügen et al., 2023). The tick microbiota varies significantly based on factors such as geographical origin, species, sex, life stages, environmental stress, tick immunity, host and blood meal (Van Treuren et al., 2015; Aivelo et al., 2019; Brinkerhoff et al., 2020; Aivelo et al., 2021; Batool et al., 2021; Duncan et al., 2022). Research has increasingly shown that changing environmental conditions can impact specific tick-associated microbes, particularly pathogens (Oechslin et al., 2017; Aivelo et al., 2019). These abiotic factors may directly influence microbiota composition and diversity by affecting microbial growth, competition and transmission (Thapa et al., 2018), or indirectly by altering tick behavior (Gray, 1991). Another important parameter is the variation in biological features between male and female ticks (Bonnet et al., 2017). While seasonal activity is similar between adult males and females (Randolph et al., 2002), females become more engorged during the nymph stage (Dusbábek, 1996), which may contribute to observed sex differences in microbiota composition (Narasimhan et al., 2021). For instance, a study conducted by Benyedem et al. (2022) on *Hyalomma* species infesting cattle across six different bioclimatic zones in Tunisia found that microbial diversity and composition vary with tick life stage and sex, particularly in *H. scupense*. The study highlighted the influence of environmental conditions and ecological niches on microbiota acquisition and expression, noting differences between domestic *H. scupense* and outdoor ticks such as *H. marginatum* and *H. excavatum* (Benyedem et al., 2022).

Shaped by internal factors like interactions among pathogenic and non-pathogenic microorganisms and responses to external perturbations, the tick microbiota becomes a dynamic microecosystem (Swei and Kwan, 2016; Cabezas-Cruz et al., 2018; Chicana et al., 2019; Wu-Chuang et al., 2021a; Aguilar-Díaz et al., 2021). For instance, Alreshidi (2020) conducted a study analyzing the bacterial community within *H. dromedarii* ticks in Saudi Arabia by sequencing the V3-V4 segment of the 16S rRNA gene. Employing this metagenomic approach, this study characterized the predominant bacterial families in *H. dromedarii* ticks from Hail city (Alreshidi, 2020), with comparable microbial community compositions observed in ticks from Hofuf city in the Eastern region (Elbir et al., 2019). Notably, endosymbiotic bacteria belonging to the genus *Francisella* were prevalent in these ticks (Elbir et al., 2019; Alreshidi, 2020). Additionally, a study conducted in Tunisia on the microbiota of *Hyalomma* species infesting cattle across various bioclimatic zones revealed similar dominant phyla, reinforcing the consistency of findings across these studies (Benyedem et al., 2022). This analysis also indicated a significant reduction in bacterial species richness in adult ticks compared to nymphs, with *Francisella* and *Rickettsia* becoming dominant in older life stages, correlating with the observed loss of microbiota diversity (Benyedem et al., 2022).

Although these studies have advanced our understanding of tick microbiota diversity and associated microbes, further analysis is required to elucidate the bacterial community assembly and microbial core components of the tick’s microbiota, as well as their functional pathways. Network analysis is a valuable tool for clarifying the complex interactions between endosymbionts, pathogens and the tick microbiota (Bonnet et al., 2017). It provides insights into the structural organization and dynamics of these microbial communities (Faust and Raes, 2012; Layeghifard et al., 2017). To illustrate the importance of network analysis in studying tick microbiota, a study by Maitre et al. (2023) used high-throughput pathogen detection and network analysis to examine the effects of *Rickettsia* pathogens on microbiota assembly in *H. marginatum* and *R. bursa* ticks. The study demonstrated that rickettsial pathogens significantly alter microbial community structure, particularly affecting the core bacterial microbiota in these tick species (Maitre et al., 2023). Furthermore, examining emergent properties, such as network robustness and connectivity, provides a deeper understanding of the behavior of complex bacterial microbiota systems (Aderem, 2005; Röttjers and Faust, 2018). Case in point, a study conducted by Piloto-Sardiñas et al. (2024) applied an *in silico* node removal approach with network analysis to simulate the absence of *Anaplasma*, assessing its impact on clustering, microbial composition and network stability over time. The removal of this tick pathogen disrupted interaction patterns and revealed the microbiota’s resilience to disturbances which highlighted *Anaplasma* as a keystone pathogen, driving dysbiosis in the *Rhipicephalus microplus* microbiota (Piloto-Sardiñas et al., 2024).

This study aimed to assess the potential influence of geographic origin and sex on the microbiota of *H. dromedarii*. Specifically, we compared the bacterial communities associated with ticks from camels in Saudi Arabia and Tunisia to evaluate geographic effects on microbiota composition. Additionally, we investigated sex-related differences to determine how biological factors shape microbial diversity. We hypothesized that geography and sex influence microbial interactions, both within microbial communities and between microbes and their tick hosts, leading to variations in overall community assembly and function beyond taxonomic distinctions alone. To address these objectives, we employed methodologies, including 16S rRNA amplicon sequencing for bacterial profiling and co-occurrence network analysis to delineate the different microbial interactions and to evaluate the persistence of co-occurring bacterial species within each microbial community.

## 2 Material and methods

### 2.1 Tick collection and identification

**FIGURE 1:**
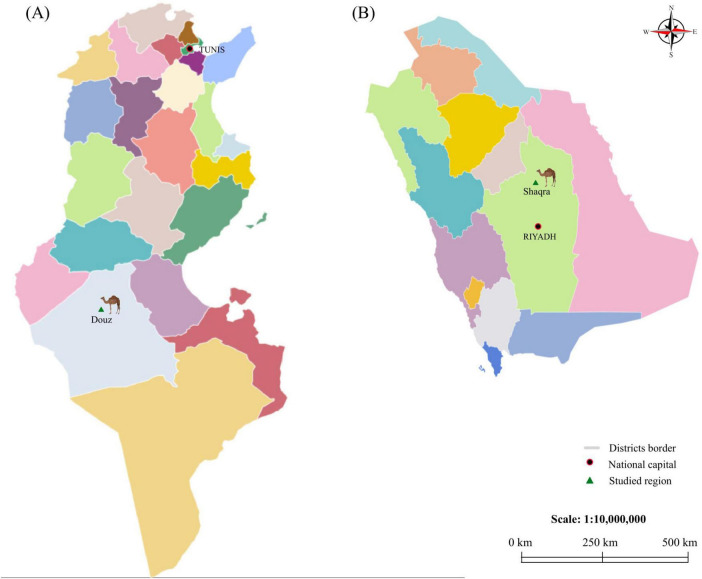
Map of Tunisia and Saudi Arabia displaying the locations of the investigated districts. **(A)** shows the map of Tunisia and **(B)** shows the map of Saudi Arabia. National capitals (Tunis and Riyadh) are indicated by red circles. The districts from which camels were collected (Douz and Shaqra) are marked with green triangles.

Between 2021 and 2022, ticks were collected from visibly healthy camels (*Camelus dromedarius*) reared in the desert of Douz, a town in the Kebili governorate in southern Tunisia (Figure 1A), and from camels in Shaqra city in the Riyadh governorate, Saudi Arabia (Figure 1B). These locations are within the same desert bioclimatic zone, characterized by an arid Saharan climate. This selection ensures a focused comparison under consistent desert environmental conditions, minimizing bioclimatic variability. Ticks were manually collected from various body sites (nostril, eye, ear, axilla, front foot, perineal area and tail). The obtained specimens were morphologically identified using taxonomic keys (Walker, 2003), and then classified according to tick species, life stage, sex and location. Each tick specimen was individually conserved in a tube containing 70% ethanol and stored at −20°C. *H. dromedarii* were selected from camels in Tunisia (*n* = 7) (Figure 1A) and Saudi Arabia (*n* = 6) (Figure 1B) based on morphological identification and sex classification. Sample sizes were balanced to ensure equal representation across locations, sex and life stage.

### 2.2 DNA extraction and amplification

Once collected, each tick was individually washed with sterile water, dried, and then crushed using an automated Tissue Lyser LT system (Qiagen, Hilden, Germany). 13 tick samples were homogenized using ATL buffer and Proteinase K solution, and DNA was extracted using the DNeasy tissue kit (Qiagen, Hilden, Germany). The eluted DNA was stored at −20°. Next, extraction efficiency was verified by PCR amplification of a 324 bp partial sequence of the mitochondrial 16S rRNA using the Techne FlexiGene Thermal Cycler (Techne Flexigene, Cambridge, UK). The PCR reaction (25 μL) included 50 ng genomic DNA, 0.4 mM of each dNTP, 0.5 μM of primers TQ16S+1F and TQ16S-2R, 0.05 U/μL Taq DNA Polymerase, 1× Taq buffer, 3 mM MgCl2, and nuclease-free water. The PCR program consisted of an initial denaturation at 94°C (8 min), followed by 10 cycles (92°C, 1 min; 48°C, 1 min; 72°C, 1.5 min), 32 cycles with hybridization at 54°C, and a final extension at 72°C (5 min). Later, species identification was confirmed on the 13 samples by Sanger sequencing of the 16S rRNA obtained amplicons using the same tick-specific primers (Black and Piesman, 1994). The sequences of each tick species were aligned assembled and corrected using ChromasPro 2.6.6 (Technelysium Pty. Ltd., Tewantin, QLD, Australia), then the corrected ticks’ sequences were submitted in GenBank^1^ to record each sequence with accession number.

### 2.3 Metagenomic 16S rRNA amplicon analysis

Sequencing of the 16S rRNA gene amplicons utilized DNA at a concentration ≥ 10 ng/μl. The procedure was outsourced to Humanizing Genomics Macrogen (Republic of Korea, KR). DNA libraries were prepared using the GS FLX Rapid Library Prep kit, from Roche Diagnostics©. A single lane of the Illumina MiSeq system was utilized to generate 251-base paired-end reads from the V4 variable region of the 16S rRNA gene in ticks, employing barcoded universal primers (515F/806R). Then, the raw 16S rRNA gene sequences obtained from tick samples were deposited at the SRA repository (Bioproject No. PRJNA1185132). Later on, the obtained 16S rRNA sequences were analyzed using the Quantitative Insights Into Microbial Ecology (QIIME2) 2023.7 pipeline. Subsequently, the raw sequences (obtained in fastq files) underwent demultiplexing, denoising, quality trimming, merging, chimera removal, and filtering using the DADA2 software (Callahan et al., 2016) integrated within QIIME2 (Bolyen et al., 2019). Eventually, amplicon sequence variants (ASVs) were aligned using MAFFT via q2-alignment plugin (Katoh et al., 2002) and employed to construct a phylogeny with FastTree2 via q2-phylogeny (Price et al., 2010). Taxonomic assignment to ASVs was accomplished using the Classify-Sklearn Naive Bayes method based on the 16S rRNA SILVA database v.138 (Yarza et al., 2014). The resulting taxonomic table collapsed at the genus level, and afterward employed for network analysis and pathway prediction.

### 2.4 Diversity indexes and taxa abundance

To assess differences in bacterial diversity within and between *H. dromedarii* tick samples from TUN and SA while also considering sexes (ML and FM) variations, alpha and beta diversity metrics were calculated using the q2-diversity plugin in QIIME2 (Bolyen et al., 2019). Alpha diversity metrics, including observed features (DeSantis et al., 2006), Pielou’s evenness index (Pielou, 1966), and Faith’s phylogenetic diversity (PD) (Faith, 1992), were analyzed within and between groups using a pairwise Kruskal–Wallis test (*p* < 0.05) in QIIME2. Beta diversity was assessed using principal coordinates analysis (PCoA) based on the Bray-Curtis dissimilarity index and analyzed with the PERMANOVA (*p* < 0.05) test in QIIME2 (Bray and Curtis, 1957). Dispersion, measuring bacterial variability between samples within the population, was calculated using the “*betadisper*” function and the “*Vegan*” package in R v.4.3.1 (R Core Team, 2024), performed in RStudio (RStudio, 2019), and an analysis of variance (ANOVA) test was used for comparison of the dispersion of the samples between the groups. Differences in bacterial taxa abundance between the groups were analyzed with the ANOVA-Like Differential Expression (ALDEx2) method (Fernandes et al., 2013) in Rstudio (RStudio, 2019). Only taxa showing significant differences (*p* < 0.05) were included in the subsequent analysis of differential taxa relative abundance. Relative abundance was measured as centered log-ratio (clr) transformation, which uses the geometric mean of the read counts in the sample, allowing for scale-free and comparable quantification between conditions (Fernandes et al., 2013). Later on, the resulting data were used to construct a heatmap using the “*heatmap.2*” function in RStudio (RStudio, 2019). Comparisons were made using Welch’s *t*-test (*p* ≤ 0.05). Clustering analysis, assessing the similarity between tick microbial samples, was performed for samples from Tunisia and Saudi Arabia and by sex, using the Jaccard coefficient of similarity and conducted with the “*Vegan*” package in R v.4.3.1 (R Core Team, 2024), implemented in RStudio (RStudio, 2019). Next, Venn diagrams were visualized, using an online tool available at http://bioinformatics.psb.ugent.be/webtools/Venn/, revealing the number of taxa shared between both countries and tick sexes.

### 2.5 Bacterial co-occurrence network analysis

Here, we employed co-occurrence network analysis to compare the architecture and node hierarchy between networks of *H. dromedarii* tick species across both countries and sex. Co-occurrence networks were constructed using taxonomic profiles at the genus level, providing a graphical representation of microbial community assemblies. In these networks, bacterial taxa are depicted as nodes, while significant correlations between taxa are represented as edges. The colors of nodes were assigned based on modularity class metric values, and the node size was proportional to the eigenvector centrality of each taxon. Significant positive (weight ≥ 0.75) or negative (weight ≤ −0.75) correlations were analyzed using the Sparse Correlations for Compositional data (SparCC) algorithm (Friedman and Alm, 2012), as implemented in the “*SpiecEasi*” R package (Kurtz et al., 2015). To further analyze network characteristics, we calculated various topological features such as the number of nodes and edges, network diameter, modularity, average degree, weighted degree, and clustering coefficient using Gephi 0.9.5 software (Bastian et al., 2009), an open-source software that transforms co-occurrence data in a graph, where these features offer insights into the stability and robustness of the bacterial community.

Additionally, the Core Association Network analysis (CAN) (Röttjers et al., 2021) was performed using the Anuran software, implemented in Python environment (Anaconda Software Distribution, 2023). This approach utilizes null models to generate random networks and assesses the properties of these networks, allowing the identification of patterns in groups of networks. CAN visualization was carried out using Gephi 0.9.5 (Bastian et al., 2009).

### 2.6 Comparative network analysis

To compare networks, a statistical estimation analysis was conducted using the package “*NetCoMi*” (Network Construction and Comparison for Microbiome Data) (Peschel et al., 2020) in R v.4.3.1 (R Core Team, 2024), and performed using RStudio (RStudio, 2019). NetCoMi offers tools for networks alignment, which involves matching nodes (microbial taxa) and edges (co-occurrence relationships) between networks based on their topological properties helping to identify corresponding features between them, even if they are not identical. To assess dissimilarities between the two networks, the Jaccard index was calculated for degree, betweenness centrality, closeness centrality and eigenvector centrality. This index evaluates the similarity between sets of “most central nodes” of networks, defined as nodes with a centrality value above the empirical 75th quartile. The Jaccard index ranges from 0 (completely different sets) to 1 (sets are equal). The two *p*-values, *P* (J ≤ j) and *P* (J ≥ j), for each Jaccard index indicate the probability that the observed value of the Jaccard index is either less than or equal to, or higher than or equal to, the Jaccard value expected at random (Real and Vargas, 1996). To assess clustering dissimilarity in networks, the adjusted Rand index (ARI) was calculated, with values ranging from − 1 to 1. Positive or negative ARI values indicate higher or lower clustering than random, respectively, with identical clustering having an ARI value of 1 and dissimilar clustering having an ARI value of 0 (Peschel et al., 2020).

### 2.7 Keystone taxa identification

Keystone taxa were identified based on three criteria: (i) ubiquity across all samples within an experimental group, (ii) eigenvector centrality higher than 0.75, and (iii) a mean relative abundance greater than the average relative abundance of all taxa within the group (Mateos-Hernández et al., 2020; Mateos-Hernández et al., 2021). The eigenvector centrality measures the influence of a node in a network, a high eigenvector score means that a node is connected to many nodes which themselves have high scores (Ruhnau, 2000). Eigenvector centrality values were derived using Gephi 0.9.5 software (Bastian et al., 2009). For each sample, the mean centered log-ratio (clr) values were calculated and plotted alongside eigenvector centrality values using GraphPad Prism version 9.0.2 (GraphPad Software, San Diego, CA, USA).

### 2.8 Network robustness analysis using node addition and removal

We evaluated the robustness of microbial co-occurrence networks by investigating the impact of node removal or addition on network connectivity. Specifically, we simulated the loss in connectivity by removing a fraction of 0.8 nodes from each network, employing both random and directed attacks. For the directed attack, we utilized three strategies: betweenness centrality, degree centrality and cascading. In the betweenness centrality approach, nodes with high betweenness centrality values were sequentially removed. Conversely, in the degree centrality approach, nodes with the highest degree centrality values were prioritized for removal. Additionally, in the cascading approach, nodes with the highest betweenness centrality values were initially removed, followed by a recalculation of betweenness centrality after each node removal. To conduct the network robustness analysis, we employed “*NetSwan*” package (Network Strengths and Weaknesses Analysis) (Lhomme, 2015) in R v.4.3.1 (R Core Team, 2024), performed using the RStudio (RStudio, 2019). Connectivity loss variability was assessed by calculating the standard error, using a threshold of 0.975.

Moreover, we conducted a node addition analysis using RStudio (RStudio, 2019), following the methodology outlined by Freitas et al. (2020). In this analysis, new nodes were randomly connected to the existing network, and the resulting changes were quantified by assessing the size of the Largest Connected Component (LCC) and the Average Path Length (APL). To enhance the precision of the network’s robustness assessment, we assessed multiple simulations with varying sets of nodes, introducing 100, 300, 500, 700 and 1,000 nodes. The outcomes were visually represented using GraphPad Prism 9.0.2 (GraphPad Software, San Diego, California, USA). Statistical significance for LCC and average path length (APL) was determined using a Wilcoxon signed-rank test, with *p*-values adjusted using the Benjamini-Hochberg (BH) method to control the false discovery rate. Additionally, bootstrapping was performed to derive confidence intervals for the variables, with significance established at a threshold of *p* < 0.05.

### 2.9 Integration of functional and taxonomic profiles for predictive analysis

To predict microbial functional traits, particularly enzymatic pathways, we utilized PICRUSt2 (Phylogenetic Investigation of Communities by Reconstruction of Unobserved States) standalone version (Douglas et al., 2020) within the QIIME2 environment. This approach leveraged various gene catalogs, including Kyoto Encyclopedia of Genes and Genomes (KEGG), Orthologs (KO), Enzyme Classification numbers (EC), Cluster of Orthologous Genes (COGs) (Kanehisa and Goto, 2000) and the MetaCyc database, to annotate major pathway categories and enable comprehensive mapping (Caspi et al., 2019). Following the output table, the taxa’s contribution to predicted metabolic pathways was investigated. To ensure robust statistical analysis, various methods were employed. Initially, alpha diversity was assessed using observed features and Pielou’s evenness metrics via the q2-diversity method in QIIME2 plugin. Differences in pathway frequency were assessed using the “*DESeq2*” package (Love et al., 2014) in RStudio in R v.4.0.3 (R Core Team, 2024), enabling the identification of statistically significant alterations in pathway abundance between the groups. This analysis produced a Volcano plot with Benjamini correlation, providing a visual representation of the significance and magnitude of pathway abundance changes. The quantification of unique and shared metabolic pathways was measured by Venn diagram using an online tool available at http://bioinformatics.psb.ugent.be/webtools/Venn/. Analyses were performed using the RStudio Integrated Development Environment (IDE) v.2023.03.0-daily+82.pro2 (RStudio, 2019).

## 3 Results

### 3.1 Morphological and molecular identification of ticks

Ticks were morphologically identified as *H. dromedarii* based on deep cervical grooves and the presence of eyes on the scutum. Adult males (*n* = 5) were distinguishable from other *Hyalomma* species by the alignment of their sub-anal plates, which are positioned outside the adanal plates. The adanal plates exhibit a characteristic shape with long, strongly curved, parallel margins. Adult females (*n* = 8) were identified by a long, tapering posterolateral spur on coxa I and a V-shaped genital aperture, which slopes gradually in *H. dromedarii*. To validate this morphological identification, *H. dromedarii* samples (*n* = 13) were screened by PCR using species-specific primers targeting the mito 16S rRNA gene. This marker was successfully validated in all samples (100%), with sequence length of 324 bp (Supplementary Figure 1). The sequences have been deposited in GenBank with accession numbers PQ871416–PQ871428. BLAST analysis revealed homology rates of 99.27–100% with the *Hyalomma dromedarii* isolate 15_camel_Abu Dhabi (GenBank accession number MZ976772), originally obtained from a camel in the United Arab Emirates.

### 3.2 Microbial diversity analysis in *Hyalomma dromedarii* ticks

**FIGURE 2:**
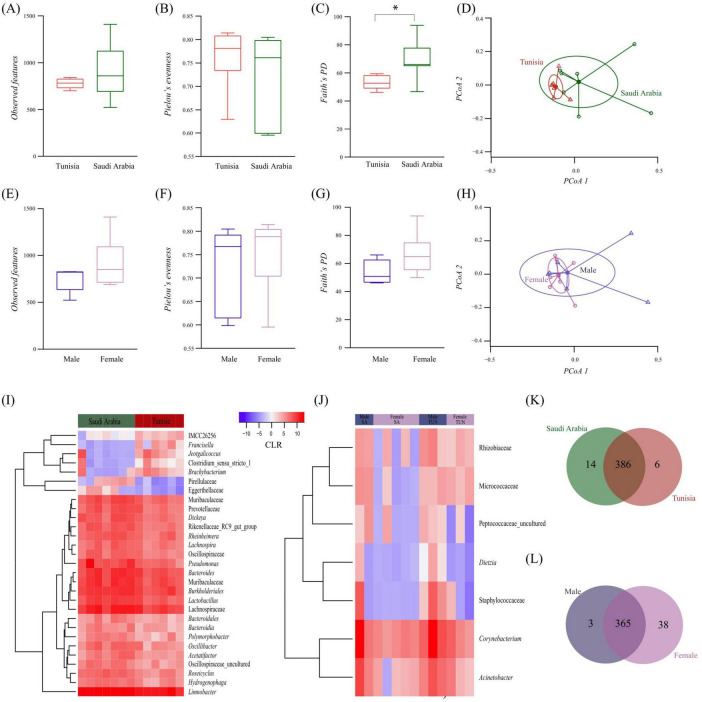
Comparison of microbial diversity and bacterial taxa abundance between *H. dromedarii* ticks from TUN and SA and across ML and FM sexes. Alpha diversity comparison using **(A)** “Observed features” index between TUN (red) and SA (green) samples, **(B)** “Pielou’s evenness” index between TUN (red) and SA (green) samples and **(C)** “Faith’s phylogenetic” index between TUN (red) and SA (green) samples (Kruskal Wallis test *p* = 0.022*). **(D)** Beta diversity comparison using the “Bray-Curtis dissimilarity” index between TUN (red) and SA (green) samples. Comparison of alpha diversity with **(E)** “Observed features” index for *H. dromedarii* from ML (dark blue) and FM (purple) samples, **(F)** “Pielou’s evenness” index between ML (dark blue) and FM (purple) samples and **(G)** “Faith’s phylogenetic” index between ML (dark blue) and FM (purple) samples. **(H)** Comparison of beta diversity with the “Bray-Curtis dissimilarity” index between ML (dark blue) and FM (purple) samples. **(I,J)** Dendrogram heatmap generated using the heatmap.2 function in RStudio (RStudio, 2019). Taxa were clustered based on relative abundance, calculated from CLR-transformed values. Each column displays CLR values for bacterial taxa per sample and group. Each row represents bacterial taxa with significant changes between the two datasets. CLR values range from −15 (blue) to 15 (red). Venn diagram comparing taxa composition between **(K)** *H. dromedarii* samples from TUN (red circle) and SA (green circle) and **(L)** *H. dromedarii* samples from FM (purple circle) and ML (blue circle) samples. Numbers indicate the total taxa in each dataset and those shared between groups.

Alpha diversity analysis, based on various metrics, revealed marginally higher observed features diversity in SA ticks compared to TUN ticks, although the difference was not statistically significant (Kruskal–Wallis test, *p* > 0.05, Figure 2A). Taxa abundance was similar between both groups (Kruskal–Wallis, *p* > 0.05, Figure 2B). However, phylogenetic analysis revealed significant diversity variance, with SA ticks exhibiting higher phylogenetic diversity than TUN (Kruskal–Wallis test, *p* = 0.022, Figure 2C). Moreover, beta diversity analysis using Bray–Curtis index indicated significant differences between SA and TUN samples (PERMANOVA, *p* = 0.048) and no significant variations in beta dispersion (ANOVA test, F = 2.35, *p* = 0.153, Figure 2D). On the other hand, the assessment within FM and ML *H. dromedarii* ticks showed slightly higher alpha diversity in females, but not statistically significant (Kruskal–Wallis test, *p* > 0.05, Figure 2E). Taxa distribution was similar between sexes (Kruskal–Wallis, *p* > 0.05, Figure 2F), with no significant differences in phylogenetic diversity (Kruskal–Wallis test, *p* > 0.05, Figure 2G). Beta diversity analysis indicated no significant sex-based clustering (PERMANOVA, *p* > 0.05; ANOVA test, F = 2.35, *p* = 0.153, Figure 2H).

Additionally, differential relative abundance analysis identified notable shifts in seven taxa, including higher prevalence of Pirellulaceae and Eggerthellaceae families in SA samples and greater abundance of *Francisella*, *Jeotgalicoccus*, *Clostridium*, *Brachybacterium* and IMCC26256 from the *Actinobacteria* group in TUN samples (Figure 2I). However, analysis by sex indicated minor variations in taxa prevalence (Figure 2J). Furthermore, compositional analysis identified 386 taxa shared between the two populations, with 14 unique to SA ticks and 6 unique to TUN ticks at the genus level (Figure 2K). Similarly, 365 taxa were shared between sexes, with 38 unique to FM and 3 unique to males ML at the genus level (Figure 2L).

### 3.3 Inference of bacterial co-occurrence patterns in networks

**FIGURE 3:**
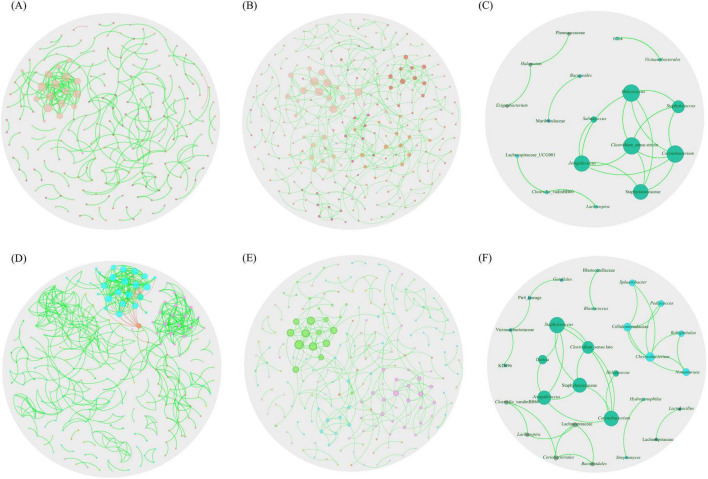
Global co-occurrence networks. Co-occurrence networks for *H. dromedarii* from **(A)** TUN and **(B)** SA. **(C)** illustrates the core network (CAN) representing shared co-occurrence interactions between the microbiota from these two countries. **(D,E)** present the co-occurrence networks for *H. dromedarii* from both sexes ML and FM, respectively. **(F)** displays the core network showing shared co-occurrence interactions between both sexes. Only nodes with at least one significant correlation are represented. Node colors are based on modularity class metric and equal color means modules of co-occurring taxa. The size of the nodes is proportional to the eigenvector centrality of each taxon. The colors in the edges represent strong positive (green) or negative (red) correlations (SparCC > 0.75 or < −0.75).

Visually, in the TUN network, one large, highly connected module was observed, composed of nodes with multiple strong positive (green) interactions. Additionally, marginal taxa with low connectivity to the main module were noted (Figure 3A). In contrast, the SA network exhibited stronger connectivity, characterized by highly interconnected taxa and the presence of two large, highly cohesive modules with robust interactions (Figure 3B). Upon analyzing co-occurrence networks, TUN network demonstrated higher values in almost all topological features (average degree, weight degree, average clustering coefficient, nodes and edges) compared to the SA network, except for positive interactions, modularity and network diameter, where the network of SA showed higher values (Table 1). The core association network (CAN) revealed 17 core associated nodes between TUN and SA networks (Figure 3C and Supplementary Table 1). *NetCoMi* was used to test dissimilarities between local network centrality measures of both networks, Jaccard index was calculated for degree, betweenness centrality, closeness centrality and eigenvector centrality (Jacc = 0, lowest similarity and Jacc = 1, highest similarity). Betweenness centrality was significantly lower than expected by random (*P* ≤ Jacc = 0.0002, Table 2), indicating a key difference in community assembly between the *H. dromedarii* networks from TUN and SA. In contrast, other centrality measures (degree, closeness, eigenvector, and hub taxa) did not show significant deviations from random expectations.

**TABLE 1:** Topological features of *H. dromedarii* networks by country and sex.

Topological features	TUN	SA	ML	FM
Connected nodes	267	257	259	203
Edges	552	365	556	251
Positives	547 (99.1%)	365 (100%)	225 (89.64%)	251 (100%)
Negatives	5 (0.906%)	0 (0%)	26 (10.36%)	0 (0%)
Modularity	0.871	0.881	0.915	0.867
Network diameter	15	17	9	10
Average degree	4.135	2.84	4.293	2.473
Weighted degree	3.308	2.367	3.214	2.075
Clustering coefficient	0.535	0.426	0.566	0.506

TUN, Tunisia; SA, Saudi Arabia; ML, male; FM, female.

**TABLE 2:** Jaccard index for *H. dromedarii* of Tunisia and Saudi Arabia.

Local centrality measures	*H. dromedarii* (TUN) vs. *H. dromedarii* (SA)		
	**Jacc^a^**	***P* (≤ Jacc)**	***P* (≥ Jacc)**
Degree	0.322	0.422574	0.644977
Betweenness centrality	0.207	0.000210***	0.999894
Closeness centrality	0.360	0.783213	0.270231
Eigenvector centrality	0.316	0.359136	0.702844
Hub taxa	0.316	0.359136	0.702844

TUN, Tunisia; SA, Saudi Arabia.

***p < 0.05.

^a^Jaccard index.

Moreover, the ML network exhibited two highly connected modules with robust positive interactions, alongside a major module featuring both positive and negative interactions (Figure 3D). In contrast, the FM network displayed a sparser configuration, with primarily positive interactions among taxa, forming a single connected module with fewer strong interactions (Figure 3E). Additionally, the CAN analysis revealed shared core interactions among taxa in both ML and FM *H. dromedarii* networks. This analysis for both sexes displayed a more intricate structure, containing 28 nodes and 33 edges (Figure 3F), compared to the networks of both countries (Figure 3C and Supplementary Table 1). Similarly, in the analysis of both countries, the Jaccard index was calculated for male and female samples. Betweenness centrality was significantly lower than expected by random (*P* ≤ Jacc = 0.000005, Table 3). However, other centrality measures (degree, closeness, eigenvector, and hub taxa) did not show significant deviations from random expectations.

**TABLE 3:** Jaccard index for *H. dromedarii* of male and female samples.

Local centrality measures	*H. dromedarii* (ML) vs. *H. dromedarii* (FM)		
	**Jacc^a^**	***P* (≤ Jacc)**	***P* (≥ Jacc)**
Degree	0.361	0.787855	0.956348
Betweenness centrality	0.179	0.000005***	0.999998
Closeness centrality	0.388	0.930155	0.095759
Eigenvector centrality	0.397	0.956348	0.061911
Hub taxa	0.397	0.956348	0.061911

ML, male; FM, female.

***p < 0.05.

^a^Jaccard index.

### 3.4 Identification of keystone taxa

**FIGURE 4:**
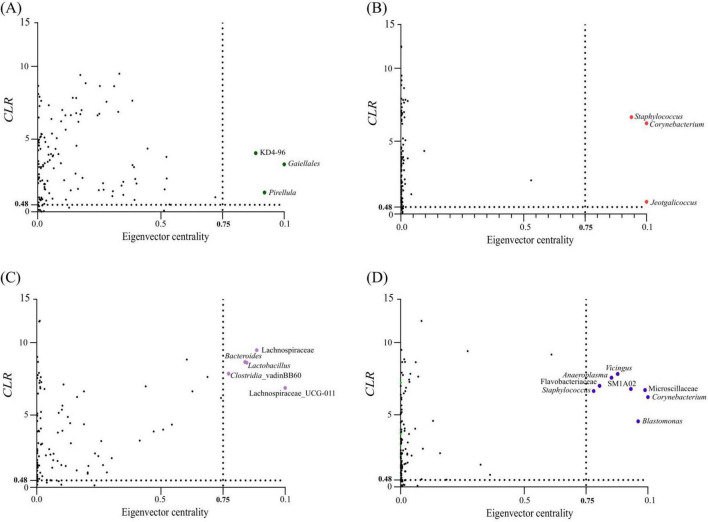
Keystone taxa in microbial communities of *H. dromedarii*. Keystone taxa of *H. dromedarii* samples are illustrated in scatter plots showing **(A)** SA, **(B)** TUN, **(C)** FM and **(D)** ML datasets. Each plot displays the mean relative abundance (CLR-transformed values) versus eigenvector centrality for ubiquitous bacterial taxa (present in all samples). The vertical dotted line indicates the eigenvector centrality cutoff value of 0.75, and the horizontal dotted line represents the average CLR value. Taxa considered keystone are those with CLR and eigenvector centrality values exceeding these thresholds. Ubiquitous taxa below the thresholds are shown as black dots, while colored dots (green, red, purple and blue) represent keystone taxa. The taxonomic identity of keystone taxa is provided for each dataset.

Keystone species, critical for the stability of microbial ecosystems, were identified based on their ubiquity, high relative abundance and high eigenvector centrality (> 0.75) in network analyses. Three taxa met these criteria for the country condition with SA *H. dromedarii* ticks showing keystone taxa belonging to the orders *Gaiellales* and *Pirellula* and the class *Chloroflexi* (KD4-96) (Figure 4A and Table 4) while TUN ticks showed keystone taxa of the genera *Staphylococcus*, *Corynebacterium* and *Jeotgalicoccus* (Figure 4B and Table 4). Additionally, a sex-specific analysis revealed distinct keystone taxa between FM and ML ticks. In FM *H. dromedarii*, the keystone species were *Lactobacillus*, Lachnospiraceae (UCG-011), Lachnospiraceae, *Bacteroides* and *Clostridia* (Figure 4C and Table 4). However, ML ticks exhibited keystone taxa such as *Vicingus*, *Corynebacterium*, *Blastomonas*, *Anaeroplasma*, *Staphylococcus*, the genus SM1A02 (*Planctomycetes*) and the families Microscillaceae and Flavobacteriaceae (Figure 4D and Table 4).

**TABLE 4:** Keystone taxa of the bacterial communities of *H. dromedarii* from TUN/SA and ML/FM group.

Condition	Keystone taxa by condition
SA	*Gaiellales* *Pirellula* *Chloroflexi* (KD4-96)
TUN	*Staphylococcus* *Corynebacterium* *Jeotgalicoccus*
FM	*Lactobacillus* *Bacteroides* *Clostridia* Lachnospiraceae (UCG-011) Lachnospiraceae
ML	*Vicingus* *Corynebacterium* *Blastomonas* *Anaeroplasma* *Staphylococcus* SM1A02 (*Planctomycetes*) Microscillaceae Flavobacteriaceae

TUN, Tunisia; SA, Saudi Arabia; ML, male; FM, female.

### 3.5 Community assembly and network robustness in *Hyalomma dromedarii* microbiota

**FIGURE 5:**
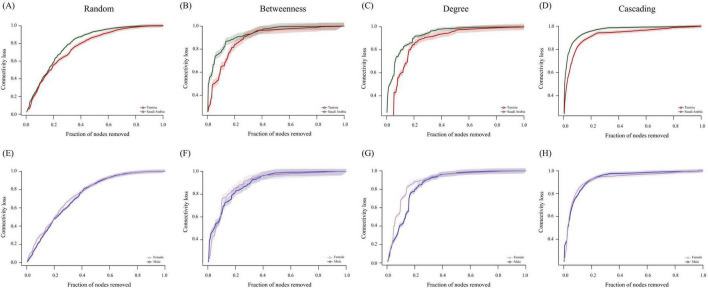
Comparison of the robustness of *H. dromedarii* microbial networks. **(A–D)** show robustness tests for networks from TUN (red) and SA (green) using random, betweenness, degree, and cascading methods, respectively. **(E–H)** display robustness tests for networks from FM (purple) and ML (dark blue) samples using the same methods. The *x*-axis represents the fraction of nodes removed from the network, while the *y*-axis indicates the fraction of connectivity loss. The solid line depicts the ratio of node removal to connectivity loss, and the shaded area represents the standard error.

The 80% connectivity loss revealed that directed attacks (betweenness, degree, and cascading) had a more significant impact on both conditions compared to random attacks (Figure 5). The TUN network demonstrated greater resilience under both random (Figure 5A) and directed attacks (Figures 5B–D), particularly under degree attacks (Figure 5C). Conversely, the SA network exhibited lower robustness under all attack types (Figures 5A–D). Specifically, degree node removal attacks caused notable changes in connectivity loss for both conditions (Figures 5C–G). Additionally, we compared the robustness of ML and FM *H. dromedarii* co-occurrence networks to determine if sex differences affect stability against node removal. The networks of ML *H. dromedarii* samples exhibited increased robustness compared to FM under directed attacks on betweenness (Figure 5F) and degree (Figure 5G). Under random attacks, ML networks also showed slightly higher robustness than FM networks (Figure 5E). However, cascading attack did not affect the robustness of ML and FM networks at a connectivity loss of 0.8, with overlapping results observed (Figure 5H).

**FIGURE 6:**
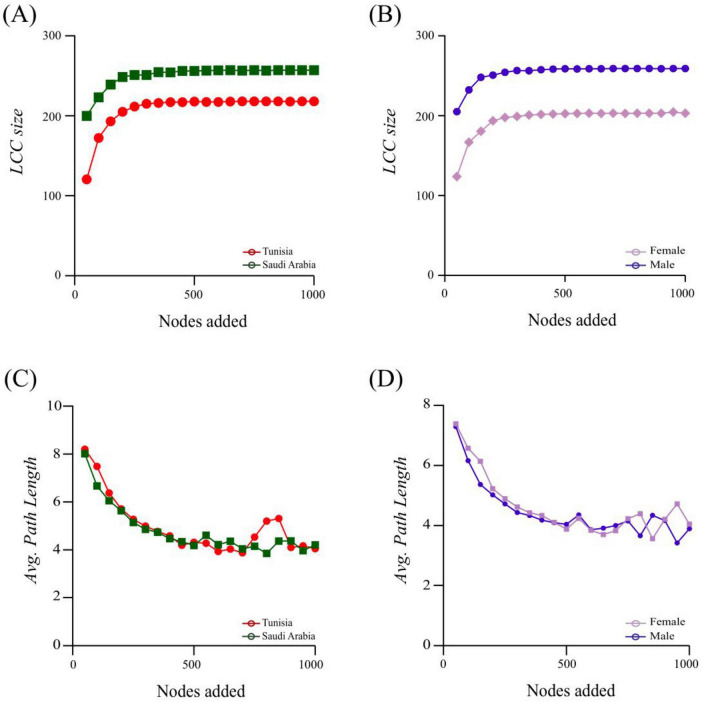
Robustness and node impact on network stability following node addition for the TUN/SA group and ML/FM group. The largest connected component (LCC) and average path length (APL) values are represented and compared between TUN and SA networks: **(A,C)** show LCC and APL values for TUN and SA networks, respectively. **(B,D)** compare the LCC and APL values between ML and FM networks. These metrics are used to evaluate network stability and robustness across different groups.

For the node addition, 1,000 nodes were introduced, and two key network properties: Largest Connected Component (LCC) and Average Path Length (APL) were assessed (Figure 6). The addition of nodes led to a greater increase in LCC for the SA network compared to TUN (Figure 6A). Both groups showed a significant enhancement in robustness with the addition of up to 200 nodes. However, LCC values began to overlap later, indicating a reduction in functional connectivity within the networks (Figure 6A). This pattern was also observed for both ML and FM samples (Figure 6B). In the APL analysis, the TUN group initially exhibited slightly higher APL values than the SA group. However, after adding 750 nodes, the APL for the TUN network increased (Figure 6C). By the 900th node, APL values for both groups had aligned. For the sex-based comparison, the FM group initially had marginally higher APL values compared to the ML group. Both groups’ APL values converged until the 950th node was added, after which the FM network increased compared to the ML network (Figure 6D). Overall, the analysis indicates that the SA network exhibited greater robustness compared to the TUN network, while the ML network was more robust than the FM network.

### 3.6 Core and central metabolic pathways in *Hyalomma dromedarii* microbiota

**FIGURE 7:**
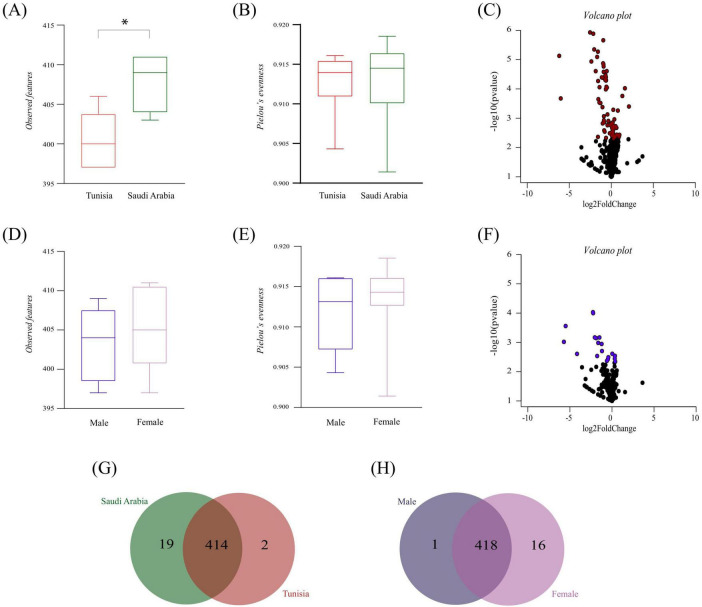
Predicted functional profile analysis. **(A,B)** Display alpha diversity metrics for the microbiota functional profiles in *H. dromedarii* from TUN (red) and SA (green) using observed features and Pielou’s evenness, respectively. **(D,E)** Show these metrics for ML (dark blue) and FM (purple) ticks. **(C,F)** Illustrate volcano plots comparing the relative abundance of functional pathways between TUN and SA, and between ML and FM samples, respectively. **(G,H)** Depict Venn diagrams of unique and shared metabolic pathways for *H. dromedarii* microbiota between country (SA vs. TUN) and tick-sex (ML vs. FM) groups. Asterisks (*) indicate statistically significant differences (*p* < 0.05).

The alterations in microbial community composition and structure were assessed to determine whether they affected the inferred functional profile of tick microbiota. A thorough analysis was conducted, comparing the composition, diversity and relative abundance of metabolic pathways in the microbiota of *H. dromedarii* ticks from both the SA and TUN group and ML and FM group. The diversity metric for observed features (Kruskal–Wallis, *p* = 0.007, Figure 7A) was significantly higher in the SA group compared to the TUN group, whereas Pielou’s evenness index showed no significant difference between these groups (Kruskal–Wallis, *p* > 0.05, Figure 7B). Moreover, a volcano plot highlighted fold changes in the relative abundance of metabolic pathways between the SA and TUN groups (Figure 7C). In the sex-specific comparison, FM ticks showed marginally higher richness than ML ticks, but this difference, as well as Pielou’s evenness index, was not statistically significant (Kruskal–Wallis test, *p* > 0.05, Figures 7D,E). Another volcano plot depicted fold changes in metabolic pathway abundance between ML and FM ticks (Figure 7F). In addition, analysis revealed both unique and shared metabolic pathways within the microbiota of SA and TUN tick groups. Specifically, 19 pathways were unique to the SA tick microbiota, including amino acid and polyamine metabolism pathways essential for protein synthesis and cellular growth, as well as carbohydrate and nucleotide biosynthesis pathways. Notably, we also identified pathways involved in antibiotic synthesis (Figure 7G and Supplementary Table 2). Nevertheless, the TUN tick microbiota exhibited two unique pathways, among which was the vitamin B6 biosynthesis pathway (Figure 7G and Supplementary Table 2). Additionally, 414 pathways were shared between the SA and TUN microbiota, primarily linked to biosynthesis, as recorded in the MetaCyc database (Caspi et al., 2019). On the other hand, in the sex-based analysis, the ML tick microbiota exhibited one unique pathway: vitamin B6 biosynthesis (Figure 7H and Supplementary Table 2). However, the FM tick microbiota revealed 16 unique pathways, cataloged in databases such as MetaCyc and KEGG (Kanehisa and Goto, 2000; Caspi et al., 2019). These pathways include significant metabolic processes, particularly those involved in amino acid and fatty acid degradation, which are essential for nutrient processing and energy metabolism. Also, several pathways were linked to the presence in environmental or gut microbiota pathways, indicating their role in microbial communities that thrive in diverse environments, including soil, gut and extreme habitats, where microbes utilize complex compounds for nutrient acquisition and detoxification (Figure 7H and Supplementary Table 3). Furthermore, both ML and FM tick microbiota shared a total of 418 pathways (Figure 7H and Supplementary Table 3), predominantly associated with biosynthesis, underscoring the metabolic capabilities essential for sustaining microbial community stability, as documented in the MetaCyc database (Caspi et al., 2019).

## 4 Discussion

The MENA region, characterized by its hyper-arid climate, serves as a crucial biogeographical link between Africa and Eurasia (Abahussain et al., 2002; Perveen et al., 2020a). Ticks, particularly *Hyalomma* spp., are widespread across Tunisia, where they are the dominant ectoparasites infesting cattle (Bouattour and Darghouth, 1996; Gharbi and Darghouth, 2014). However, research in Tunisia has primarily focused on the identification, epidemiology and conventional control of livestock and zoonotic tick-borne pathogens, leaving a knowledge gap concerning the microbiota of *H. dromedarii*, especially in southern Tunisia (Benyedem et al., 2022). Similarly, Saudi Arabia continue to face persistent challenges with tick infestations in livestock, notably camels (Perveen et al., 2021b). A longitudinal study conducted from 2019 to 2020 confirmed the year-round presence of *H. dromedarii* on camels in many Saudi Arabian regions (Perveen et al., 2020a). Despite this, research on the microbiota community associated with this tick in the country remains limited. Furthermore, geographical location and environmental factors are known to influence the composition of an organism’s microbiota (Perveen et al., 2022a). Therefore, we expect microbial adaptation to the desert environment, potentially resulting in distinct microbiota compositions in *H. dromedarii* ticks (Perveen et al., 2022a). In this study, we aimed to elucidate the effects of geographical origin (TUN and SA) and tick sexes (ML and FM) on the composition and stability of *H. dromedarii* microbiota. We hypothesized that these factors could influence microbial interactions, leading to distinct community profiles and functional variations. The present results suggest that the microbiota of *H. dromedarii* is potentially influenced by both geographic origin and sex, with distinct bacterial communities observed between populations in Saudi Arabia and Tunisia, as well as sex-specific microbiota patterns, underscoring the influence of environmental and biological factors on community structure and metabolic functions.

Our findings are consistent with previous studies on the *H. dromedarii* tick microbiota, particularly concerning the distribution of bacterial phyla. In alignment with earlier reports on *H. anatolicum* and *H. dromedarii* (Adegoke et al., 2020; Perveen et al., 2020b), we observed a predominant presence of *Proteobacteria*, followed by Firmicutes and *Actinobacteria*. Notably, Elbir et al. (2019) reported that *Proteobacteria* constituted over 98% of the microbiota in *H. dromedarii* ticks collected from Hofuf city in Saudi Arabia, followed with low prevalence of Firmicutes and *Actinobacteria*. Our study’s phyla distribution corroborates these findings, as well as those of Thapa et al. (2018), who identified *Proteobacteria* as the most abundant phylum in *Ixodes scapularis* ticks across various temperature ranges in the USA (Thapa et al., 2018). Additionally, *Proteobacteria* was identified as the dominant phylum in *I. ricinus* ticks found on sheep in Northern Italy (Carpi et al., 2011). Collectively, these results show that *Proteobacteria* is a common phylum present across different tick species (Perveen et al., 2020b).

Besides, in our study, it was not entirely surprising that we detected the genus *Francisella* at high relative abundance in the TUN group compared to the SA group. In fact, *Francisella* is commonly found as an endosymbiont in various tick species, and its prominence in Tunisian *Hyalomma* spp. ticks likely results from the interplay between the microbiota community and the ecological adaptations related to the tick’s restricted diet (Benyedem et al., 2022). Known for their involvement in tick nutrition (Hodosi et al., 2022), *Francisella* has also been associated with the camel ticks *H. dromedarii* in Saudi Arabia (Elbir et al., 2019; Alreshidi, 2020), Egypt (Ghoneim et al., 2017), UAE (Perveen et al., 2020b), Palestine (Ravi et al., 2019) and recently, Kenya (Khogali et al., 2024). The prevalence of endosymbionts like *Francisella* aided by suitable microclimates and high vertebrate host density from extensive camel farming, likely supports the resilience of camel tick populations in harsh environments (Perveen et al., 2022a). This endosymbiont has been identified in multiple tick species in mutualistic associations (Bonnet et al., 2017). However, phylogenetic similarities between pathogenic and mutualistic *Francisella* strains suggest frequent shifts from non-pathogenic and pathogenic forms (Narasimhan and Fikrig, 2015; Bonnet et al., 2017). Nonetheless, the co-occurrence of non-pathogenic and pathogenic bacteria may not always result in genetic transformations (Greay et al., 2018), suggesting that multiple factors could influence pathogenicity in tick microbiota.

Moreover, highly prevalent genera such as *Pseudomonas* were detected in both countries (TUN and SA), aligning with findings from previous studies. Indeed, *Pseudomonas* presence in camel ticks has been previously documented across various tick species such as *R.* (*Boophilus*) *microplus*, *I. ovatus* and *I. persulcatus* ticks (Carpi et al., 2011; Estrada-Peña et al., 2018; Aivelo et al., 2019). This genus is commonly found in the sandy soils of Saudi Arabia (Eida et al., 2018), and it has been suggested that ticks can acquire these bacteria through openings such as the mouth, spiracles or anal pore (Narasimhan and Fikrig, 2015). Consequently, this study cannot conclusively determine whether *Pseudomonas* is an environmental acquisition or true members of the tick microbiota. Furthermore, in the sex-related analysis, our results revealed the presence of relatively abundant genera, such as *Acinetobacter* and *Corynebacterium*, in both ML and FM tick microbiota. Notably, *Staphylococcus* was also identified during the core-associated network (CAN) analysis and as a keystone species, highlighting its pattern within the group’s networks. These findings align with previous studies that reported *Acinetobacter*, *Corynebacterium* and *Staphylococcus* as among the most abundant genera detected in the microbiota of *H. dromedarii* collected from sheep (Carpi et al., 2011). Our results revealed a positive correlation between the keystone taxa *Staphylococcus* and *Corynebacterium* within the *H. dromedarii* microbiota network of ML and TUN ticks. These keystone taxa significantly shape microbiota structure and function in specific spatial or temporal contexts (Banerjee et al., 2018; Wu-Chuang et al., 2021b). For instance, previous studies have shown that four keystone bacteria (*Pseudomonas*, *Ralstonia*, *Acinetobacter* and *Bradyrhizobium*) maintained the functional diversity of *I. scapularis* microbiota under high-temperature stress (Wu-Chuang et al., 2021a; Wu-Chuang et al., 2021c). Despite temperature-induced shifts in taxonomic profiles, this core set of keystones remained consistent across various temperatures (Wu-Chuang et al., 2021a; Wu-Chuang et al., 2021c), underscoring their role in microbial community stability and resilience. In addition, another recent study conducted by Khogali et al. (2024) identified *Acinetobacter* spp., *Pseudomonas* spp., and *Corynebacterium* spp. in *H. dromedarii* ticks from camels in Kenya (Khogali et al., 2024). While some research suggests these bacteria may be contaminants, others propose they are environmental bacteria acquired and maintained by ticks throughout their life cycle (Hernández-Jarguín et al., 2018; Díaz-Sánchez et al., 2019; Li et al., 2022). Of note, Mohamed et al. (2021) reported the presence of these genera in camel blood. This supports the hypothesis of their circulation between camel blood and ticks. Additionally, these bacterial genera have been identified at tick bite sites (Zhang et al., 2023), indicating their potential role in modulating inflammation and influencing pathogen transmission through the host’s response to tick bites (Bernard et al., 2020). Concretely, the potentially pathogenic *Staphylococcus* and *Corynebacterium* were similarly detected in various tick species, including *R. microplus*, *R. sanguineus*, *I. ricinus*, *I. holocyclus*, *A. tuberculatum*, *I. ovatus*, *I. persulcatus*, *H. flava*, *H. rufipes*, *H. aegyptium*, *H. marginatum* and *H. excavatum* (Elbir et al., 2019). This also raises the question that these prevalent bacterial genera may encode functions associated with tick survival and reproduction, warranting further investigation. Furthermore, our observation of *Staphylococcus* in the microbiota of *H. dromedarii* suggests the potential presence of pathogenic species, such as *Staphylococcus* aureus, within the animal populations that this tick species parasitizes (Perveen et al., 2022b). Indeed, by identifying these different key microbial species and understanding how environmental and biological factors shape microbial communities, future research can explore strategies to manipulate the tick microbiota, potentially reducing pathogen transmission and enhancing the effectiveness of tick control methods.

The concept of robustness, defined as a network’s resistance to disturbances, can be effectively elucidated through percolation theory (Cohen et al., 2011), which provides insights into information flow among network nodes (Röttjers and Faust, 2018). This methodology has been validated as a predictive tool for ecosystem behavior, positioning network robustness as a potential indicator of microbial community resilience across various animal taxa, including arthropods (Estrada-Peña et al., 2020a; Mateos-Hernández et al., 2021) and mammals (Mahana et al., 2016). By linking network properties to the dynamic behaviors of microbial communities, we uncover promising diagnostic applications (Maitre et al., 2023). In this study, we applied percolation theory to evaluate network robustness by assessing the loss of connectivity through metrics such as degree, cascading, betweenness and random attacks. Our findings revealed that the impact of node removal differed significantly, particularly affecting the SA and FM networks. Specifically, targeted degree attacks highlighted nuanced differences in network structure and resilience strategies between the microbiota associated with the country and sex group. The TUN microbiota demonstrated greater resilience to targeted removals, indicating a network structure that better withstands the loss of species. Also, the ML microbiota exhibited greater robustness against degree and other-directed attacks, suggesting a distinct arrangement of essential functions compared to the FM microbiota. This emphasizes the ecological significance of network structure in microbial communities, with implications for understanding ecosystem stability, managing microbial health and conserving biodiversity. Beyond changes in community assembly and stability, microbial functional profiles are shaped by biotic and abiotic factors (Maitre et al., 2024). The SA group exhibited greater functional richness and evenness than the TUN group, indicating broader metabolic capabilities. Similarly, the FM group displayed higher functional richness than the ML group. Our metabolic analysis uncovered distinct functional profiles between both the country (TUN and SA) and sexes (ML and FM) groups, with variations in genes related to carbohydrate and amino acid metabolism. These metabolic distinctions may signify adaptations to different environmental conditions or host preferences, potentially affecting tick feeding, reproduction and pathogen transmission (Maitre et al., 2024). While some metabolic pathways were shared, each group exhibited unique pathways, underscoring functional differences. This functional redundancy in the *H. dromedarii* microbiome across both countries and sexes aligns with findings by Estrada-Peña et al. (2020b), who reported similar patterns in tick microbiomes. This redundancy, defined by the presence of identical genes or functional categories across diverse microbes, may reflect an evolutionary strategy to maintain essential functions and provide ecological benefits (Obregón et al., 2019; Estrada-Peña et al., 2020a,b). Additionally, it likely enhances microbiome stability under stress, minimizing functional disruptions. Moreover, differences in taxonomic and functional microbiomes between tick species, and between females and males of the same species, suggest that tick gut microbiota specialize in response to the life cycle and fitness requirements of their hosts (Obregón et al., 2019). In this sense, further studies are needed to elucidate the relationship between metabolic pathways, gene expression, and microbiota modulation. Understanding these pathways could inform tick control strategies aimed at disrupting key metabolic functions to reduce tick survival and tick-borne disease transmission.

## 5 Conclusion

Our study provides valuable insights into the influence of geographic origin and sex on the microbiota of *H. dromedarii*, revealing distinct bacterial communities associated with ticks from Saudi Arabia and Tunisia, as well as sex-specific microbiota patterns. These findings highlight the interplay between environmental and biological factors in shaping microbial communities, influencing both community structure and metabolic functions. Our approach has unveiled the complexity of microbial interactions and the persistence of co-occurring bacterial species within these communities. However, one of the study’s limitations is the relatively small sample size, which may restrict the generalizability of our findings. While small sample sizes can offer valuable preliminary insights, they are not sufficient to draw robust conclusions. We acknowledge this limitation and emphasize the need for future studies with larger population sample sizes to confirm our conclusions and further investigate the effects of geographic and sex-related factors on tick microbiota. Additionally, environmental factors, such as the health and nutritional status of the host camels, may also influence tick microbiota composition and warrant further exploration.

## Data Availability

The datasets presented in this study can be found in online repositories. The names of the repository/repositories and accession number(s) can be found below: https://www.ncbi.nlm.nih.gov/, PRJNA1185132.

## Footnotes

1
http://www.ncbi.nlm.nih.gov/genbank/

## Supplementary material

Supplementary Figure 1 Molecular identification of *Hyalomma dromedarii* ticks using polymerase chain reaction (PCR) targeting the mitochondrial 16S rRNA gene. PCR products (324 bp) were resolved on 1.5% agarose gels stained with ethidium bromide. Lane M: 100 bp DNA ladder; Lanes 1–7 present 16S rRNA amplicons from *H. dromedarii* samples collected from SA. Whereas, lanes 8–13: 16S rRNA amplicons from *H. dromedarii* samples collected from TUN; Lane T-: negative control.

Supplementary Table 1 Core association network (CAN) topological features of *Hyalomma dromedarii*.

Supplementary Table 2 The list of shared and unique pathways of functional profile analysis between TUN and SA.

Supplementary Table 3 The list of shared and unique pathways of functional profile analysis between ML and FM.
