# Supplementary figures and images for: Microbial community variations in adult Hyalomma dromedarii ticks from single locations in Saudi Arabia and Tunisia
Source: Front Microbiol. 2025 Feb 11;16:1543560. doi: 10.3389/fmicb.2025.1543560 (PMC11850374; doi:10.3389/fmicb.2025.1543560)

**M**

**1**

**2**

**3**

**4**

**5**

**6**

**7**

**8**

**9**

**10**

**11**

**12**

**13**

**T<sup>-</sup>**

324 bp →

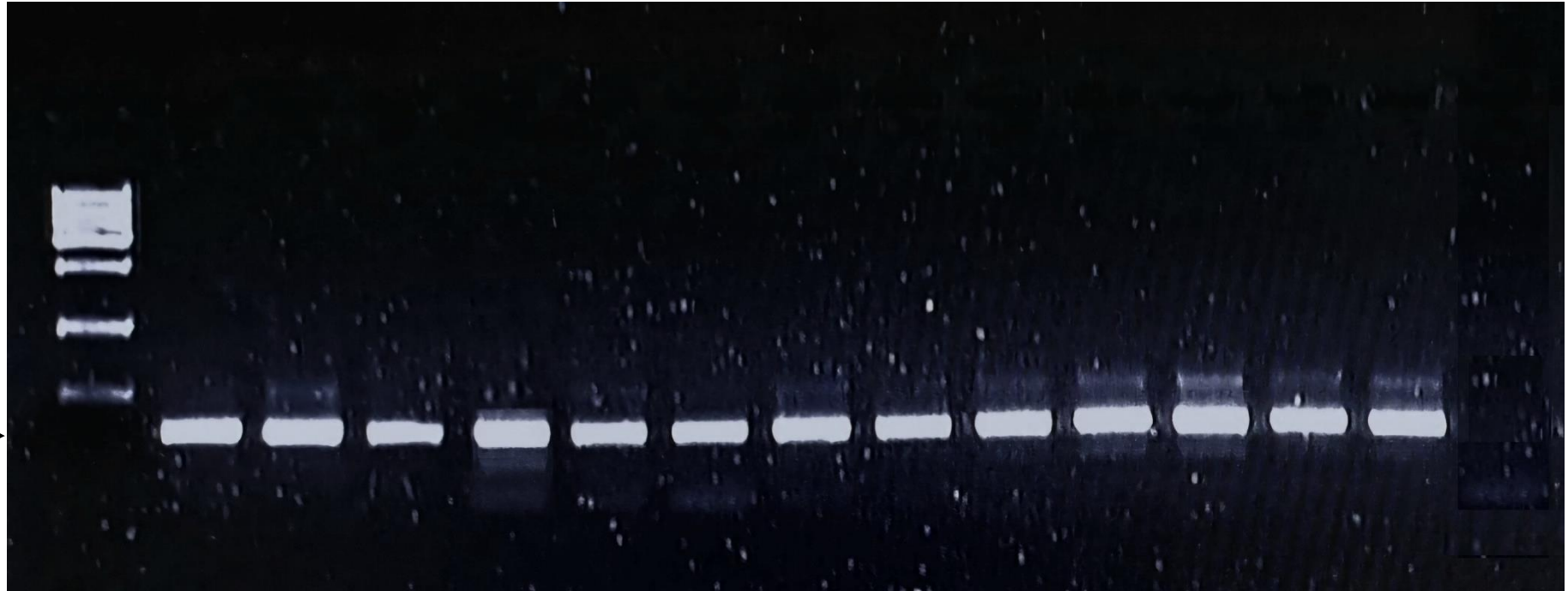

Supplement: Supplementary Figure 1 — Molecular identification of Hyalomma dromedarii ticks using polymerase chain reaction (PCR) targeting the mitochondrial 16S rRNA gene. PCR products (324 bp) were resolved on 1.5% agarose gels stained with ethidium bromide. Lane M: 100 bp DNA ladder; Lanes 1–7 present 16S rRNA amplicons from H. dromedarii samples collected from SA. Whereas, lanes 8–13: 16S rRNA amplicons from H. dromedarii samples collected from TUN; Lane T-: negative control. [file Image_1.pdf]
