# Supplementary material for: Microbial community variations in adult Hyalomma dromedarii ticks from single locations in Saudi Arabia and Tunisia
Source: Front Microbiol. 2025 Feb 11;16:1543560. doi: 10.3389/fmicb.2025.1543560 (PMC11850374; doi:10.3389/fmicb.2025.1543560)
Supplement: Supplementary Table 1 — Core association network (CAN) topological features of Hyalomma dromedarii. [file Table_1.docx]

**Supplementary Table S1. Core Association Network (CAN) topological features of *Hyalomma dromedarii***

| **Topological features** | **TUN/SA** | **ML/FM** |
| --- | --- | --- |
| Connected nodes | 17 | 28 |
| Edges | 20 | 33 |
| Positives | 20 (100%) | 33 (100%) |
| Negatives | 0 (0%) | 0 (0%) |
| Modularity | 0.485 | 0.75 |
| Network diameter | 3 | 3 |
| Average degree | 2.353 | 2.357 |
| Weighted degree | 2.069 | 2.035 |
| Clustering coefficient | 0.625 | 0.57 |

Abbreviations : TUN : Tunisia ; SA : Saudi Arabia ; ML : Male ; FM : Female
